# Chemical Characterization, Stability and Sensory Evaluation of Sicilian Extra Virgin Olive Oils: Healthiness Evidence at Nose Reach

**DOI:** 10.3390/foods13132149

**Published:** 2024-07-06

**Authors:** Claudia Lino, David Bongiorno, Rosa Pitonzo, Serena Indelicato, Manfredi Barbera, Gabriella Di Gregorio, Domenico Pane, Giuseppe Avellone

**Affiliations:** 1 ATeN Center, Università di Palermo, Viale delle Scienze, 90128 Palermo, Italy; claudia.lino@unipa.it (C.L.); rosa.pitonzo@unipa.it (R.P.); 2 Dipartimento di Scienze e Tecnologie Biologiche Chimiche e Farmaceutiche, Università di Palermo, Via Archirafi 32, 90123 Palermo, Italy; serena.indelicato@unipa.it; 3 Manfredi Barbera & figli S.p.a., Via E. Amari, 55/A, 90139 Palermo, Italy

**Keywords:** phenolic compound, extra virgin olive oil (EVOO), sensory evaluation, olive cultivars, liquid chromatography mass spectrometry, health benefits

## Abstract

The aim of this study was to assess the nutraceutical qualities of extra virgin olive oil (EVOO) samples obtained from three Sicilian olive *cultivars*: *Nocellara*, *Biancolilla*, and *Cerasuola.* We also evidenced the relationship among biophenols, base parameters and panel test scores, and evaluated the stability of the biophenols in EVOO. The assessment also took into consideration variations in olive harvesting periods and the influence of four different milling methods. A statistical analysis of the collected data revealed that the cultivar and harvesting period were the primary factors influencing the bio-phenol content, while the milling methods employed did not significantly affect the levels of biophenols in the oils. The panel test results were also illuminating as they were strongly related to the cultivar and polyphenol content. Following the criteria outlined in EC Regulation 432/2012, we selected three samples, each representing one of the cultivars, which exhibited the highest bio-phenol content to evaluate the biophenol stability during a time span of 16 months.

## 1. Introduction

The “Mediterranean Diet” represents a model of a healthy and sustainable dietary pattern that incorporates the traditional eating habits of the Mediterranean region. It emphasizes the consumption of plant-based foods, including cereals, legumes, fruits, vegetables, and extra virgin olive oil (EVOO), along with a moderate intake of animal-based products such as meat, dairy products, and fish. Among the foods in the Mediterranean diet, EVOO is considered a functional food due to its healthy properties. As such, it combines nutritional and pharmaceutical properties [1,2,3]. EVOO contains mainly triglycerides (98–99%) that represent the saponifiable fraction, and unsaponifiable constituents (1–2%), which include hydrocarbons, phytosterols, fat-soluble vitamins, pigments, aliphatic and triterpene alcohols, and polyphenols [4,5]. Numerous studies have demonstrated the positive nutritional effects of a diet based on the moderate consumption of extra virgin olive oil, primarily attributable to its bio-phenol content. These compounds possess antioxidant, anti-inflammatory, anti-cancer, antimicrobial, antiviral, hypoglycemic, hepatic, cardiac, and neuroprotective properties, making EVOO highly regarded in the diet due to the presence of several bioactive compounds [6,7]. Bio-phenols are part of the so-called “nutraceutical components” [1,8] and constitute the largest group of secondary plant metabolites with recognized health qualities. The terms “polyphenols” or “bio-phenols” encompass chemical species containing an aromatic ring substituted with one or more hydroxyl groups. The content and composition of bio-phenols vary among different sources such as fruits, oil, leaves, and waste, as well as within the same source (such as pulp and stone in olives). Furthermore, the hydrophilic phenol content in EVOO can be influenced by various agronomic factors, including the olive cultivar, ripeness level (linked to the harvesting period), climate, soil conditions, irrigation, technical processes employed during oil separation, as well as storage conditions [5,9,10].

Recognizing the valuable effects of a healthy diet [6,7], the European Food Safety Authority (EFSA) allows for health recognition based on the bio-phenols in olive oil, with reference to the food claims summarized in CE Regulation 432/2012, which categorizes olive oil as a functional food acknowledging that “Olive oil polyphenols contribute to the protection of blood lipids from oxidative stress”. This regulation permits the use of a specific health claim on olive oil labels, stating that it should contain a minimum of 5 mg of hydroxytyrosol and its derivatives (such as oleuropein complex and tyrosol) per 20 g of olive oil [11]. This concentration corresponds to a minimum total phenolic compound content in EVOO of no less than 250 mg/kg [12].

Olive oil contains several classes of bio-phenols, including phenolic alcohols, phenolic acids, flavonoids, lignans, phenylpropanoids, and secoiridoid derivatives [1,13,14]. The main phenolic alcohols found in olive oil are hydroxytyrosol (3,4-DHPEA) and tyrosol (p-HPEA). Although these compounds are typically present in low concentrations in fresh olive oil, their levels increase during storage due to the hydrolysis of secoiridoids [1]. The latter are a group of compounds that are usually glycosylated and derive from the secondary metabolism of terpenes. Secoiridoids, found exclusively in the Oleaceae family that includes *Olea europaea* L., are compounds characterized by the presence of elenolic acid in its glycosidic or aglyconic form. Oleuropein and ligstroside are the main secoiridoids in olive fruits [12].

During the olive oil production process, the crushing and malaxation stages bring oleuropein and ligstroside into contact with endogenous β-glucosidases, leading to their conversion into their respective oleuropein aglycone (3,4-DHPEA-EA) and ligstroside aglycone forms (p-HPEA-EA). However, these aglycones exist as multiple isomers due to keto-enol tautomerism, are highly unstable and can only be observed under very specific conditions. Within the olive oil matrix, they undergo transformation into more stable closed and monoaldehydic forms or alternatively into open-ring di-aldehydic forms. The di-aldehydic forms derived from oleuropein and ligstroside aglycones are known as oleocanthal (p-HPEA-EDA, the dialdehyde form of decarboxymethyl-elenolic acid bound to tyrosine) and oleacein (3,4-DHPEA-EDA, the dialdehyde form of decarboxymethyl-elenolic acid bound to 3,4-DHPEA), respectively [1,14].

Other bio-phenols found in olive oils include phenolic acids such as p-hydroxybenzoic acid and its derivatives (e.g., gallic, protocatechuic, syringic, and vanillic acids), as well as p-hydroxycinnamic acid and its derivatives (e.g., p-coumaric, caffeic, and ferulic acids). Additionally, minor phenolic compounds such as lignans (e.g., pinoresinol and 1-acetoxypinoresinol) and flavones (e.g., luteolin, diosmetin, and apigenin) are present [5].

Bio-phenols have been determined as a whole by NIR, and also quantified independently using liquid chromatography coupled with high-resolution mass spectrometry (UPLC-HRMS). This latter approach has multiple applications [15,16] and can be employed for both screening and quantification purposes. One significant advantage of HRMS is its ability to conduct the retrospective analysis of acquired data, enabling the determination of a wide range of molecules, including nutraceuticals, lipids, contaminants, antioxidants, polymers, surfactants, and proteins [14,15,16,17,18,19,20,21,22]. Our study evaluated 36 samples of olive oil belonging to three differing cultivars (*Nocellara*, *Biancolilla* and *Cerasuola*) and harvested in two differing periods with four differing milling systems to quantify polyphenols and to determine their claim values. The same samples were subjected to the conventional determination of the quality parameters of EVOOs (free fatty acids content, fatty acid profile, spectrophotometric indices K232, K270 and ΔK), and finally were tested by a trained panel to obtain the corresponding perceptual scores. The aim was to evidence any relationship among biophenols, base parameters and the panel test scores, and to evaluate the stability of the biophenols in EVOO.

## 2. Materials and Methods

### 2.1. Chemicals

Methanol and water (LC–MS grade) were purchased from J.T. Baker™ (Deventer, The Netherlands); acetic acid 100% for LC-MS LiChropur™ was purchased from Merck (Darmstadt, Germany), and diethyl ether, absolute ethanol, phenolphthalein, sodium hydroxide, potassium iodide, chloroform, acetic acid (glacial), starch salt, sodium thiosulphate (normex) and iso-octane were purchased from Carlo Erba reagent (Cornaredo, Italy).

All the following chemical standards were purchased from Merck (Darmstadt, Germany): 3,4-DHPEA, p-HPEA, p-coumaric acid, ferulic acid, apigenin, luteolin, p-HPEA-EDA, 3,4-DHPEA-EDA, ethyl gallate were phyproof^®^ Reference Substances; gallic acid was certified reference material TraceCERT^®^.

### 2.2. Extra Virgin Olive Oil Samples Collection

**Scheme 1:**
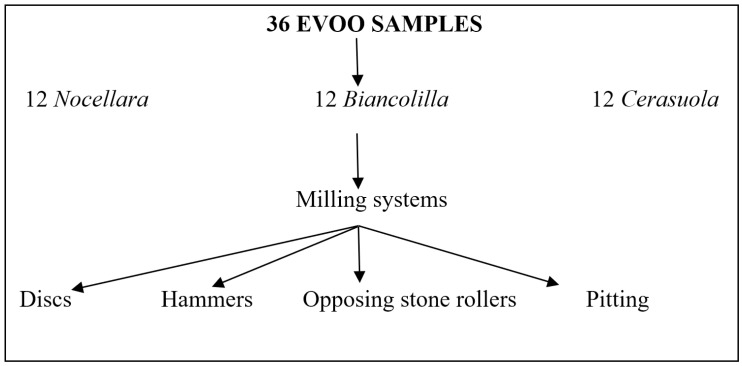
Milling procedure adopted for producing the 36 EVOO samples.

The samples of EVOO analyzed in this study were provided by “Manfredi Barbera & Figli S.p.A.”, a renowned Sicilian company founded in 1894 by Lorenzo Barbera, known internationally for its olive oil production. For the experimental process, a newly developed mill was utilized, employing four different milling systems: discs, hammers, opposing stone rollers, and pitting. Each system exerts distinct mechanical energy on the olives, resulting in variations in the olive paste temperature. A total of 36 samples of EVOO produced from olive trees belonging to three Sicilian cultivars, namely Nocellara, Biancolilla, and Cerasuola, were analyzed. Specifically, the analysis included 12 Nocellara samples, 12 Biancolilla samples, and 12 Cerasuola samples, as illustrated in Scheme 1 below.

Since it is known that the olive ripening and harvesting period has an influence on the chemical composition of olive oils [10], the olives from these cultivars were harvested during two different periods: the first harvest took place in October 2022, and the second in November 2022. All collected samples were promptly processed upon arrival.

In our study, the significant number of independently produced olive oil samples (36) allowed for the observation of distinct trends and attributes.

### 2.3. Extraction of Free Fatty Acids

The extraction for the following determination of free fatty acids (cold method) was performed according to International Olive Council IOC methods [23].

### 2.4. Spectrophotometric Determination with UV

This determination was performed on a Thermo Fisher (Bremen, Germany): Genesys 150Uv-Visible Spectrophotometer, diluting 0.25 g of olive oil with iso octane in a 25 mL volumetric flask and measuring the absorbance at 232 nm, 266 nm, 270 nm and 274 nm according to IOC methods [24].

### 2.5. Determination of Total Polyphenols by Near Infrared Spectroscopy

The measurements were performed according to the following procedure: a 40 × 8 mm clear vial was filled with 1 mL of oil and placed inside the sample holder of the instrument before starting the measurement. If the oil sample was cloudy, it was filtered using a 0.45 μm syringe filter before filling the vial. The instrumentation used was an Antaris II FT-NIR Analyzer Thermo Fisher Spectroscopic in the 10,000 to 4000 cm^−1^ range, with a resolution: 8 cm^−1^. The Number of co-mediated scans was 32, with a collection time of 24 s.

The instrument performance was verified before use with Thermo Scientific ValPro™ system qualification 1.0 software using an internal validation wheel with NIST traceable standards to ensure the consistency of the equipment’s photometric response. An internal gold flag was used for background collection.

The chemometric processing was performed using the Thermo Scientific TQ Analyst™ 3.0.65 (Service Pack 12) chemometric software package. All data were centered on the mean value and then converted to the respective second derivative spectra prior to the development of the calibration models. For this purpose, a Norris derivative with a 9-point segment and no gap was used. Derivatization is an option typically used to remove the multiplicative dispersion phenomena common in NIR measurements. Scattering generally does not contribute to providing information relevant to the measurement of interest. The first derivative normalizes the spectral offset while the second derivative normalizes the slope of the baseline. After data derivation, calibrations were constructed using Stepwise Multiple Linear Regression (SMLR) and Partial Least Squares (PLS) regression models.

### 2.6. Extraction and Determination of Phenolic Compounds

The phenolic compounds in the EVOO samples were extracted and quantified following the established literature [25]. The extraction procedure involved mixing 2 g of oil with 5 mL of methanol/water (80:20 *v*/*v*). The samples were then vortexed for 1 min, subjected to ultrasonic treatment in a bath for 15 min at room temperature, and centrifuged at 5000 rpm for 25 min at 20 °C. The resulting hydroalcoholic phase was recovered and filtered using PTFE filters with a pore size of 0.45 μm. Before the injection, 20 μL of the internal standard (IS), ethyl gallate (10 mg/10 mL), was added to 1 mL of each extract [26,27,28].

### 2.7. Standard Solution of Phenolic Compounds

**Figure 1:**
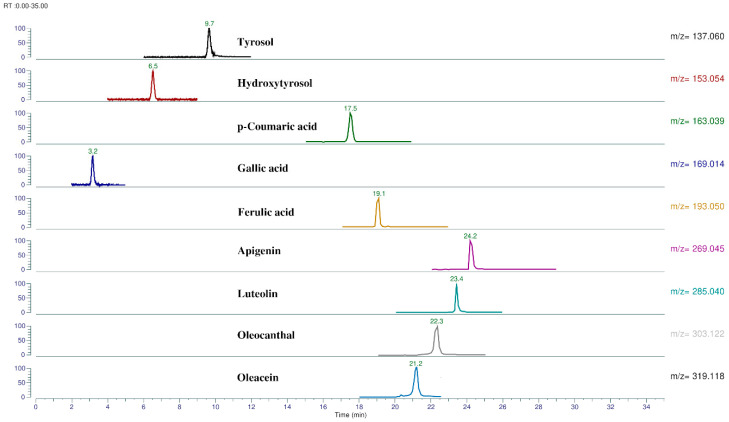
Chromatograms of pure standards adopted for quali-quantitative purposes.

**Figure 2:**
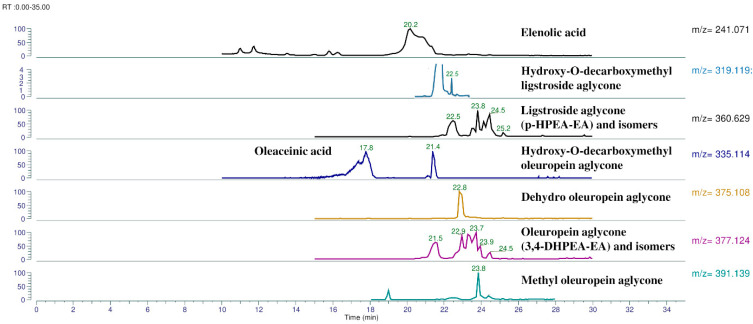
Chromatograms displaying the analytes identified in the Nocellara cultivar using the disc milling system.

In this study, we detected and quantified a total of 17 phenolic compounds, and their chemical classes are presented in Table 1. However, individual standards were not available for each phenolic compound quantified. To overcome this limitation, we employed relative quantification methods. Elenolic acid, hydroxy-O-decarboxymethyl ligstroside aglycone (HDLA), and p-HPEA-EA with its monoaldehydic isomeric forms were quantified and expressed as p-HPEA-EDA equivalent. Similarly, hydroxy-O-decarboxymethyl oleuropein aglycone (HDCOA), oleaceinic acid, dehydro oleuropein aglycone (DHOA), 3,4-DHPEA-EA with its monoaldehydic isomeric forms, and methyl oleuropein aglycone (MOA) were quantified and expressed as 3,4-DHPEA-EDA equivalent. For the remaining phenolic compounds, standard stock solutions were prepared using methanol/water (80:20 *v*/*v*) as the solvent and stored at −20 °C. Calibration curves were constructed at four concentration levels. The working solutions of bio-phenols at various concentration ranges were prepared by mixing aliquots of each stock solution, as follows: p-HPEA ranged from 2 to 0.5 mg/L, 3,4-DHPEA ranged from 3 to 0.1 mg/L, p-coumaric acid ranged from 1.5 to 0.1 mg/L, gallic acid ranged from 0.5 to 0.03 mg/L, ferulic acid ranged from 0.6 to 0.05 mg/L, apigenin ranged from 2.5 to 0.3 mg/L, luteolin ranged from 3 to 0.5 mg/L, p-HPEA-EDA ranged from 450 to 30 mg/L, and 3,4-DHPEA-EDA ranged from 300 to 10 mg/L. Figure 1 displays a chromatogram of a standard mixture solution, while a chromatogram showing the identified analytes in a real sample is presented in Figure 2.

### 2.8. Determination of Phenolic Compounds by UPLC-HESI-HRMS

Phenolic compounds were identified using a UPLC-Q Exactive Orbitrap-HRMS constituted by coupling a Dionex (Sunnyvale, CA, USA) Ultimate 3000 liquid chromatograph and a Thermo Fisher Scientific™ (Bremen, Germany) Q Exactive™ Plus Hybrid Quadrupole-Orbitrap™ Mass Spectrometer. The ion source employed was a heated electrospray ionization (HESI) ion source. A Dr. Maisch Reprosil Saphir (Ammerbuch, Germany) 100 C18 column (150 × 2.0 mm, 5 µm) equipped with a precolumn was used for chromatographic separation. The elution gradient, at a flow rate of 400 µL/min, consisted of mobile phase A, namely water with 0.1% acetic acid, and mobile phase B, namely methanol. The elution conditions were as follows: 2 min isocratic elution with 5% (B); 3 min gradient elution up to 10% (B); 11 min gradient elution up to 25% (B); 12 min gradient elution up to 95% (B); 2 min isocratic elution with 95% (B); 2 min gradient to 2% (B); and 3 min isocratic elution with 5% (B). The injection volume was 1 μL. The parameters for the Q Exactive Orbitrap system were set as follows: spray voltage −3.0 kV, capillary temperature 250 °C, auxiliary gas heater temperature 300 °C, sheath gas flow rate 30 au, and auxiliary gas flow rate 15 au. Mass detection was performed using two acquisition modes: negative-ion mode full scan with a mass resolving power of 35,000 FWHM at *m*/*z* 200, an AGC target of 1 × 106, a maximum injection time of 200 ms, and a scan range of 100–1500 *m*/*z*; and targeted selected ion monitoring t-SIM with a quadrupole isolation window of 1.0 *m*/*z* and a resolution power of 35,000 FWHM at *m*/*z* 200, following methods established in the literature [28,29,30,31]. The phenolic compounds identified in the EVOO samples, along with their retention times (RT) and accurate masses, are reported in Table 1.

### 2.9. Determination of Fatty Acids Profile by GC/MS

Sample preparation for the analytical determination of FAMEs by means of GC/MS involved dissolving approximately 0.1 g of oil in a 5 mL screw-top test tube containing 100 µL of 2 N KOH solution in methanol and 1 mL of n-hexane. The solution was vigorously shaken for 2 min. Phase separation: in the aqueous phase, the glycerin is solubilized; meanwhile, in the hexane phase, the supernatant contains the methyl esters useful for the analysis.

GC analyses were conducted using a Thermo Fisher ISQ LT mass spectrometer equipped with a Trace 1310 GC. The experimental parameters included a 30 m, 0.25 mm i.d. and a 0.25 µm film thickness Zebron ZB-5MS (Thermo Fisher) column, with helium as the carrier gas at a flow rate of 1 mL/min. The oven temperature was programmed as follows: an initial temperature of 120 °C was held for 3 min, ramped at 10 °C/min to 300 °C. Subsequently, the temperature was ramped at 10 °C/min to reach 350 °C and held for 5 min, resulting in a total run time of 32 min. A 1 µL injection with a 1:25 split ratio was made, and the injector temperature was set to 200 °C. The identification of FAMEs in olive oil samples utilized the Supelco 37 Component FAMEs mix standard (Supelco, Bellefonte, PA, USA). The mass spectrometer, equipped with an Electron Ionization (EI) source set at an ionization potential of 70 eV, operated in the Selected Ion Monitoring (SIM) mode, monitoring ions at *m*/*z* 55, *m*/*z* 57, *m*/*z* 69, *m*/*z* 74, and *m*/*z* 87, and in full scan mode with a scan range of *m*/*z* 50–400. The transfer line and ion source temperatures were both set at 290 °C. FAMEs were identified based on retention times and a spectral comparison with the NIST 2015 Mass Spectral Library. The fatty acid quantities were expressed as the relative percentages of the total fatty acid content. Peak integration was performed using Xcalibur™ 3.0.6 3software (Thermo Scientific™, Waltham, MA, USA).

### 2.10. Sensory Evaluation and Panel Test

The sensory evaluation of the oils was performed in a single-blind manner, using alphanumeric casual codes to identify each sample to avoid positive and negative biases according to IOC methods [31,32]. The panel was composed of 1 panel leader and 8 panel testers, and was periodically recalibrated according to the IOC methods [32].

### 2.11. Stability Evaluation of EVOOs

The polyphenol content in three selected EVOOs showing the highest content of biophenols per cultivar was monitored for 16 months of aging.

The EVOO samples were stored in brown bottles at a controlled temperature (15–25 °C) and opened just prior to analyses. The following polyphenol determination was carried out using near-infrared spectroscopy, as detailed in Section 2.5.

## 3. Results

A total of 36 samples of EVOO produced from Sicilian cultivars *Nocellara*, *Biancolilla*, and *Cerasuola* were analyzed to detect and quantify the 17 phenolic compounds listed in Table 2. To comply with the health claim specified in EC Regulation 432/2012, it is crucial to evaluate all possible compounds containing the 3,4-DHPEA moiety.

These compounds, as reported in the literature [12,29,30], are listed in Table 2 and identified as compound 2, 10, 12, 15, 16, and 17. To establish the quantitative limit for the health claim regarding the expression of p-HPEA, Covas et al. considered the collective contribution of p-HPEA and its derivatives in calculating the 5 mg/20 g of olive oil. These compounds, also reported in Table 2 as compounds 1, 9, 11, 13, and 14, align with the previous literature [33,34,35,36,37].

To determine the claim values, the amounts of the 12 phenolic compounds were, therefore, summed. These include p-HPEA-EA and its derivatives (p-HPEA-EA with its monoaldehydic isomeric forms, HDLA, p-HPEA-EDA, and oleaceinic acid); p-HPEA and its aglycones (3,4-DHPEA-EDA, HDCOA, dehydrooleuropein aglycone, 3,4-DHPEA-EA with its monoaldehydic isomeric forms, MOA); and elenolic acid.

The free p-HPEA and 3,4-DHPEA detected in the 36 EVOO samples were, as expected, in low concentrations.

The *Nocellara* samples showed a 3,4-DHPEA content ranging from 0.25 to 4.47 mg/kg, and a p-HPEA content ranging from 0.68 to 1.61 mg/kg. The main phenolic compound contents were by far the following: 3,4-DHPEA-EDA, 3,4-DHPEA-EA, elenolic acid, PHPEA-EA and PHPEA-EDA, which represent, respectively, 11%, 19%, 36%, 17% and 16% of the total polyphenol content. Smaller quantities were detected for the following secoiridoids: HDCOA, HDLA, oleaceinic acid, DHOA, and MOA, which represent about 1% of the total polyphenol content. The calculated claim values of the *Nocellara* oil samples ranged from 9.4 to 15 mg/20 g of olive oil. The *Nocellara* oil sample obtained with the disc milling system showed the highest claim value.

The *Biancolilla* samples showed a 3,4-DHPEA content ranging from 0.37 to 2.07 mg/kg, and a p-HPEA content ranging from 0.64 to 0.91 mg/kg. Also, for *Biancolilla* oils, the main phenolic compound contents were by far 3,4-DHPEA-EDA, 3,4-DHPEA-EA, Elenolic acid, PHPEA-EA and PHPEA-EDA, which represent, respectively, 14%, 21%, 26%, 18% and 19%. The calculated claim values of the *Biancolilla* oil samples ranged from 9.70 to 14.11 mg/20 g of olive oil. For the samples of this cultivar, the disc milling system gave the highest claim value.

The *Cerasuola* samples showed a 3,4-DHPEA content ranging from 0.58 to 4.14 mg/kg, and a p-HPEA content ranging from 0.68 to 1.55 mg/kg. The most representative bio-phenols even for the *Cerasuola* oils were 3,4-DHPEA-EDA, 3,4-DHPEA-EA, elenolic acid, PHPEA-EA and PHPEA-EDA, which represent, respectively, 11%, 25%, 20%, 26% and 16%. Interestingly, for *Cerasuola* samples, the most represented biophenol was no longer elenolic acid but PHPEA-EA. The calculated claim values of the *Cerasuola* oil samples ranged from 13.87 to 17.78 mg/20 g of olive oil. Analogous to the previous results, the oil obtained with the disc milling system showed the highest claim value.

The fatty acid compositions of the olive oil samples were evaluated by GC/MS analysis of the corresponding fatty acid methyl esters (FAMES, see Table 3). From this point of view, the sampled oils showed a similar profile, as similar values (ranging from 0.13 to 0.28 g/100 g) were found for acidity. Although these measurements suggest the similar maturation of the sampled olives, two differing harvesting periods were chosen.

The conventional quality parameters for olive oils, with free acidity peroxide values and the spectrophotometric extinction coefficients at 232 nm, 266 nm, 270 nm and 274 nm, are reported in Table 4. The extinction coefficients at 232 nm (K232) and 270 nm (K270) are indicators of primary and secondary oxidation, while acidity is symptomatic of triacylglycerol degradation.

Table 4 shows that all these parameters, well within the IOC limits for extra virgin olive oils, are also, on average, very close for all the cultivars.

## 4. Discussion

### 4.1. Statistical Analysis

Various types of analyses were carried out to determine if a trend could be identified from the acquired data, including principal component analysis (PCA) and subsequent linear discriminant analysis (LDA). All statistical evaluations were conducted using TIBCO Statistica 14.1.25 software. The results showed that no data segregation could be observed through PCA.

The first evidence obtained from our study is related to the organoleptic characteristics determined by a trained panel test. It is worth recalling that these are single-blind analyses, and that the panel testers were not aware of the cultivar or any other feature of the tested oils, which were simply identified by an alphanumeric casual code, as requested by the IOC [34]. The blind test assigns only score points to unknown samples. These scores are linked to each sample (and to the information as cultivar, milling method etc.) only a second time, avoiding biases in judging the oils. At this stage, the panel testing alone is still not capable of distinguishing among the three differing EVOO cultivars, even if it could give some hint in this direction. Indeed, the following statistical re-elaboration of the average scores of fruitiness, bitterness and spiciness for the *Nocellara* cultivar showed that these are significantly different from the others, as revealed by a *t*-test at a 95% confidence level. In particular, the average scores of fruitiness were as follows: 4.84, 5,47 and 6.52 for the *Biancolilla*, *Cerasuola* and *Nocellara* oils, respectively. For the positive attribute of the spiciness, the scores were, respectively, 3.69, 4.30 and 5.2; meanwhile, for the attribute of bitterness, the scores were, respectively, 3.40, 4.20 and 4.4. A more detailed evaluation of the sensory data also evidences a marked correlation between these two parameters, which therefore could be easily grouped as a single variable that is potentially capable of discriminate between oils of differing cultivars well.

**Figure 3:**
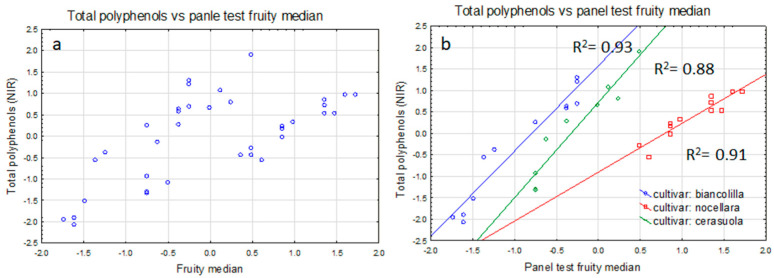
(**a**) Plot of fruity median vs. total polyphenols (NIR) without categories (cultivars); (**b**) plot of fruity median vs. total polyphenols (NIR) with categories and linear regression results.

The correlations between the collected variables, however, do not end here; indeed, an interesting correlation between the total phenols (measured by NIR) and sensory scores (mostly with the fruity medians) is evidenced. This correlation is not immediately evident (Figure 3a) since it is cultivar dependent (see Figure 3b).

This trend suggests that the bio-phenol content has a foreseeable effect on the sensory scores; in particular, higher contents of bio-phenols are related to higher organoleptic scores. On the other hand, this correlation could be exploited in another way: once the cultivar is known, the panel test could estimate the corresponding bio-phenol level based on the organoleptic performances, or vice-versa.

Looking at Figure 3b, it is also evident that the *Nocellara* cultivar stands on its own, and for an established polyphenol content, *Nocellara* oils evidence higher panel scores. In particular, the spicy and fruity notes are best perceived in *Nocellara* oils with respect to the *Cerasuola* and *Biancolilla* cultivars.

**Figure 4:**
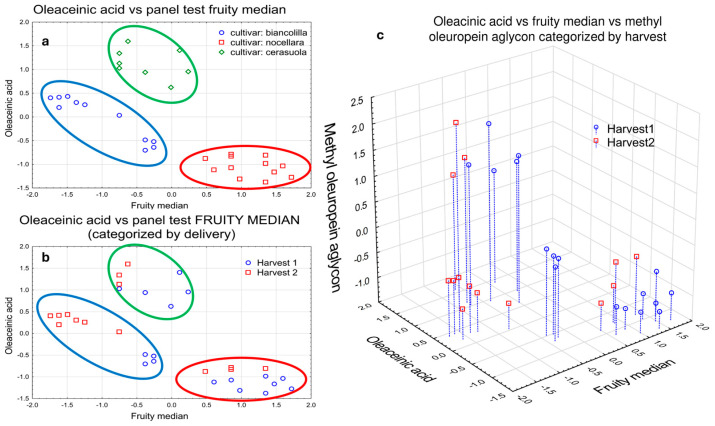
(**a**) Scatterplot of oleaceinic acid against fruity median, categorized by cultivar and (**b**) categorized by harvesting; (**c**) 3D Scatterplot of oleaceinic against fruity median and methyl oleuropein aglycon categorized by harvesting period.

By exploiting the fruitiness scores and the oleaceinic acid levels, it was possible to almost discriminate between oils of differing cultivars, avoiding the use of multivariate projection techniques, as shown in Figure 4a. Again, looking at the same graph but grouping the points by harvesting period, it is evident that samples of the same cultivar and the same harvesting period are grouped with the others, with lower values of fruitiness and higher levels of free acid for samples harvested in the second epoch (corresponding to a more mature drupe Figure 4b).

This is also well evidenced in a 3D projection that also takes into account the MOA levels and further amplifies the distance among cultivars (Figure 4c).

A perusal of this graph also evidences that all the values characterizing the *Cerasuola* and *Biancolilla* oils are similar to the *Nocellara* oil values when considering an early harvesting. In order to find the polyphenolic features most impacted by the cultivar expression, we performed LDA, which allowed us to distinguish the oil based on the cultivar at a *p* = 0.05 level. The analysis was intentionally performed with a reduced number of discriminant variables (5) that were selected by means of a stepwise procedure among all the polyphenol levels (continuous variables). The procedure also involved external validation, leaving out 15% of the samples to validate the model created using the remaining 85%. The classification results (both model and validation samples) led to a single sample misclassification (97% accuracy), as shown in Table 5.

The variables used in the model were the following: ferulic acid, MOA, oleaceinic acid, ligstroside aglycon, and apigenin.

**Figure 5:**
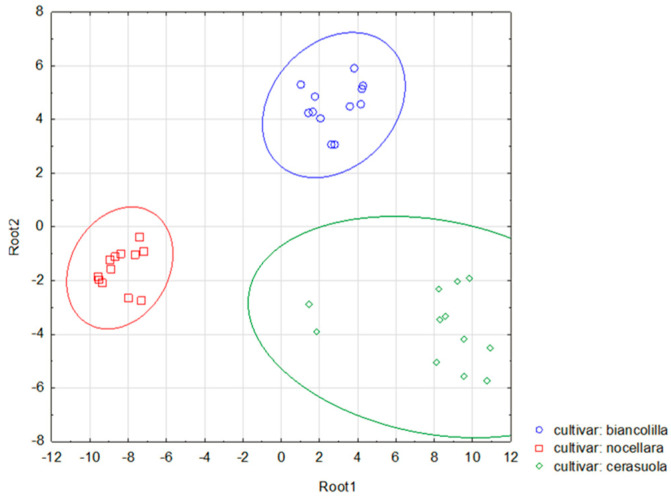
Scatterplot of canonical scores of olive oil categorized by cultivars, reporting 95% confidence ellipses and significance *p* and f values related to the classification.

The test for the significance of the distance and the 95% probability ellipses show the good separation allowed by the features selected during the LDA analysis (Figure 5 and Table 6).

It is also noteworthy that, by using all the quantitative data that were chromatographically determined to perform the LDA analysis, the stepwise procedure leads always to the individuation of these same features (ferulic acid, MOA, oleaceinic acid, ligstroside aglycon and apigenin) belonging to the polyphenol class of substances. In other words, the prevision model remains the same. This implies that polyphenol levels are strictly related to the olive cultivars and, as previously evidenced for triacyclglycerols [1,38], are also capable of discriminating among oils belonging to differing cultivars.

Since the effects of the ripening and harvesting period on the oil quality parameters have been evidenced in the literature [39], we tried to perform LDA based on the harvesting period to find the most discriminating variables capable of evidencing the difference between the oils harvested in differing months. In this case, we further reduced the number of variables used in the classification to show the ones most strongly related to the harvesting period.

The stepwise procedure was performed using all the continuous variables at our disposal, i.e., the organoleptic scores, which were reduced to three variables using a stepwise regression. The model was created again using 85% of the randomly selected samples and then using the remaining 15% for the external validation of the model. In the first instance, we determined whether polyphenol-related variables could be successfully used to discriminate between harvesting periods. The results (Table 7a) show that polyphenols alone can explain the differences in oils based on their harvesting time with excellent accuracy. In this case, the variables chosen for the model are as follows: gallic acid, DHPEA and oleacein.

The results reported in Table 7b refer to a LDA based on all the continuous variables at our disposal. Also, in this case, the excellent (100%) accuracy of the discrimination and validation are evidenced, as well as significant f and p scores. The variables selected for the model are the following: gallic acid, K232, and DHPEA.

Finally, the trial we performed to evidence differences between the fresh oils obtained with differing crushing methods was not successful.

This indicates that these technological aspects play a secondary role compared to the balsamic harvest time or the selected *cultivar* in determining the overall bio-phenol content of the olive oil.

This first pioneering study could not take into consideration multiple harvesting seasons that, for several reasons (solar radiation, temperatures, rainfall, winds, humidity), are known to also have a strong influence on the chemical quality of olive oils [10,40,41]. It has also to be taken into consideration that it is not easy to guarantee the stability of a panel test among differing seasons (but possible) since it requires periodic recalibration, otherwise leading to less reliable results. Nonetheless, the strongest correlation between the sensory parameters and polyphenol levels that were reported to be the least variable among the quality indices in differing seasons [41] suggests that both sensory evaluation and the polyphenol content could represent robust variables in a multi-season evaluation of quality EVOOs.

### 4.2. EVOOs Stability Evaluation

**Figure 6:**
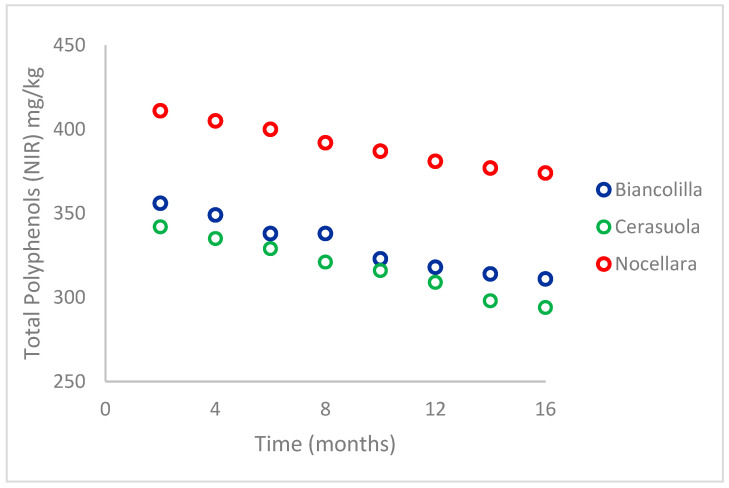
Total polyphenols (NIR) in olive oil vs. time in months.

The polyphenol content in the three selected EVOOs that showed the highest content of bio-phenols per cultivar was followed during sixteen months of aging. As clearly shown in Figure 6, a decrease in the polyphenol levels was observed. The claim values, however, remained well within the requested limits [10]. This last finding shows that good-quality EVOOs with an elevated content of bio-phenols can survive prolonged storage. In our case, they lost only about 10–15% of their original bio-phenol content during the 16-month stability evaluation. The storage conditions adopted within this study were mild, with only glass brown bottles being used as a precaution to avoid the samples becoming impaired, while the temperature was controlled to avoid causing excessive thermal stress to the stored EVOOs, being maintained at under 25 °C. These conditions, within the reach of most people and storage facilities, can be easily replicated in order to preserve the quality of “healthy” EVOOs for at least one season.

## 5. Conclusions

The analysis of phenolic compounds in 36 samples of Italian (Sicily) extra virgin olive oil confirmed their compliance with the EC Regulation 432/2012 health claim, underscoring the potential health benefits associated with these compounds.

Our study revealed significant variations in the phenolic composition among the three studied cultivars, namely *Nocellara*, *Biancolilla*, and *Cerasuola*, indicating that the choice of cultivar plays a pivotal role in determining the overall biophenol content and potential health benefits of the olive oil. Moreover, the harvesting period was found to be another influential factor affecting the phenolic composition of the olive oils. Discriminant analysis based on the harvesting time successfully classified the samples, highlighting the importance of considering the timing of harvest for optimizing the bioactive phenolic content of the final olive oil product. Notably, certain specific phenolic compounds, such as MOA, oleaceinic acid, ligstroside aglycon, and apigenin, showed significant variations among different cultivars and harvesting periods, suggesting the influence of genetic and environmental factors on their production.

It was not possible (even it is advisable) to follow this study during multiple harvesting seasons, but these findings still provide valuable insights for producers and consumers alike as they emphasize the significance of selecting the appropriate cultivar and optimizing the harvest time to enhance the nutritional and health-promoting properties of the olive oil. It appeared also noteworthy that the results of the panel testing whose scores were well correlated with the total polyphenol contents of olive oils, and once provided with more information, could provide inform us about the olive oil cultivars. On the other hand, the milling systems did not evidence remarkable effects on the quality of the EVOO samples, nor did they influence their sensorial properties.

Oil stability, in terms of the polyphenol content, was evaluated in a time range of 16 months, during which the polyphenol content was reduced but remained within the range of compliance with EC Regulation 432/2012.

This is positive news, as it evidences that a properly stored, good-quality olive oil can still be healthy after several months (at least 1 year). While it was not possible to perform a more comprehensive evaluation that also took into consideration volatile components, the results of this work are encouraging and suggest that by further exploring the specific properties of individual phenolic compounds and optimizing cultivation practices, the olive oil industry can increase the quality of products that promote health and well-being for consumers worldwide.

## Figures and Tables

**Table 1:** Phenolic compound identification based on retention time (RT), quasi molecular ion, theoretical and experimental *m*/*z*.

Phenolic Compounds	RT (min)	Molecular Formula	Theoretical *m*/*z* [M − H]^−^	Experimental *m*/*z* [M − H]^−^	Calculated Errors (Δppm)
**Phenolic Alcohols**					
p-HPEA	9.7	C_8_H_10_O_2_	137.06080	137.05954	1.26
3,4-DHPEA	6.5	C_8_H_10_O_3_	153.05572	153.0591	1.81
**Phenolic Acids**					
p-Coumaric acid	17.6	C_9_H_8_O_3_	163.04007	163.03848	1.58
Gallic acid	3.2	C_7_H_6_O_5_	169.01425	169.01311	1.14
Ferulic acid	19.1	C_10_H_10_O_4_	193.05063	193.04970	0.93
**Flavonoids**					
Apigenin	24.3	C_15_H_10_O_5_	269.04555	269.04523	0.32
Luteolin	23.4	C_15_H_10_O_6_	285.04046	285.04004	0.42
**Secoiridoids**					
Elenolic acid	20.1	C_11_H_14_O_6_	241.07176	241.0761	0.63
p-HPEA-EDA	22.3	C_17_H_20_O_5_	303.12380	303.12344	0.35
3,4-DHPEA-EDA	21.1	C_17_H_20_O_6_	319.11871	319.11823	0.49
HDLA	22.4	C_17_H_20_O_6_	319.11871	319.11887	0.48
HDCOA	21.4	C_17_H_20_O_7_	335.11363	335.11392	0.88
Oleaceinic acid	17.8	C_17_H_20_O_7_	335.11363	335.11392	0.88
p-HPEA-EA with its monoaldehydic isomeric forms	22–26	C_19_H_22_O_7_	361.12928	361.12967	1.09
DHOA	22.9	C_19_H_20_O_8_	375.10854	375.10873	0.52
3,4-DHPEA-EA with its monoaldehydic isomeric forms	20–25	C_19_H_22_O_8_	377.12419	377.12442	0.61
MOA	23.9	C_20_H_24_O_8_	391.13984	391.14032	1.22

**Table 2:** Content of phenolic compounds (mg/kg) in 12 *Nocellara*, 12 *Biancolilla* and 12 *Cerasuola* olive oil samples (EVOO).

N.	Phenolic Compounds	12 *Nocellara* Samples (mg/kg)			12 *Biancolilla* Samples (mg/kg)			12 *Cerasuola* Samples (mg/kg)		
		Range	Mean	Sd	Range	Mean	Sd	Range	Mean	Sd
1	p-HPEA	0.68–1.61	1.15	0.34	0.64–0.90	0.74	0.10	0.68–1.54	0.82	0.24
2	3,4-DHPEA	0.25–4.47	2.02	1.73	0.37–2.07	1.01	0.63	0.58–4.14	1.50	1.03
3	p-Coumaric acid	0.034–0.56	0.33	0.13	0.10–0.20	0.14	0.04	0.05–0.26	0.09	0.06
4	Gallic acid	0.010–0.03	0.02	0.006	0.003–0.025	0.01	0.0006	0.001–0.02	0.01	0.01
5	Ferulic acid	0. 14–0.24	0.20	0.03	0.045–0.079	0.06	0.01	0. 02–0.12	0.06	0.02
6	Apigenin	0.08–0.65	0.24	0.14	0.31–0.55	0.45	0.08	0. 12–0.45	0.31	0.08
7	Luteolin	0.14–1.39	0.87	0.27	0.93–1.38	1.14	0.14	0.50–1.05	0.76	0.17
8	Elenolic acid	74.44–279.87	204.45	55.50	130.89–162.74	149.09	8.07	116.16–195.66	156.51	27.28
9	p-HPEA-EDA	74.69–115.59	90.11	13.66	80.73–140.38	110.78	22.56	94.48–155.25	128.25	21.65
10	3,4-DHPEA-EDA	48.76–87.81	65.18	11.74	61.95–99.88	76.49	12.70	63.84–113.64	87.91	16.61
11	HDLA	1.17–2.74	1.96	0.60	1.95–8.82	4.67	2.68	0.89–6.92	2.66	1. 57
12	HDCOA	0.41–0.83	0.54	0.13	0.63–1.52	1.06	0.34	0.21–2.87	0.79	0.72
13	Oleaceinic acid	0.05–0.97	0.55	0.32	1.10–2.97	2.22	0.73	3.17–9.91	5.03	2.14
14	3,4-DHPEA-EA and monoaldehydic isomeric forms	60.37–181.20	128.19	44.71	111.22–136.56	119.89	9.04	144.07–223.11	195.79	22.95
15	DHOA	1.61–4.53	2.75	0.77	3.25–4.27	3.89	0.32	3.89–7.36	5.16	1.18
16	p-HPEA-EA and monoaldehydic isomeric forms	53.08–228.41	116.84	49.71	52.38–174.80	109.17	47.75	153.87–250.69	206.11	23.45
17	MOA	1.36–1.83	1.52	0.16	1.45–2.10	1.78	0.21	1.95–3.07	2.65	0.32

**Table 3:** Fatty acid methyl esters (reported as relative percentages) determined in the olive oil samples.

	Biancolilla				Cerasuola				Nocellara			
	Min	Max	Mean	Sd	Min	Max	Mean	Sd	Min	Max	Mean	Sd
Palmitate	9.56	14.77	13.05	2.16	10.74	15.00	11.93	1.34	11.38	15.04	13.60	1.04
Palmitoleate	0.25	1.33	0.97	0.44	0.53	1.19	0.75	0.22	0.66	1.39	1.01	0.21
Eptadecanoate	0.06	0.19	0.13	0.05	0.08	0.20	0.12	0.04	0.11	0.19	0.15	0.03
Eptadecenoate	0.07	0.30	0.22	0.09	0.09	0.36	0.18	0.08	0.15	0.36	0.26	0.06
Stearate	2.53	2.96	2.67	0.12	2.69	3.23	2.86	0.14	2.74	3.09	2.88	0.11
Oleate	68.10	76.01	70.99	2.89	68.45	74.69	72.86	1.78	67.86	73.89	70.40	1.62
Linoleate	9.05	10.65	9.87	0.57	8.06	9.74	9.13	0.55	8.89	10.70	9.45	0.54
Arachidate	0.47	0.49	0.48	0.01	0.47	0.50	0.47	0.01	0.47	0.49	0.47	0.01
Linolenate	0.59	0.70	0.67	0.03	0.61	0.75	0.66	0.04	0.62	0.74	0.69	0.04
Eicosenoate	0.27	0.46	0.33	0.07	0.27	0.40	0.36	0.04	0.25	0.38	0.32	0.04
Lignocerate	0.06	0.07	0.06	0.00	0.06	0.06	0.06	0.00	0.06	0.07	0.06	0.00

**Table 4:** Conventional quality indices for olive oils.

	Acidity (%)	K232	K266	ΔK
*Biancolilla*	0.195	1.586	0.111	0.098
std. deviation	0.04	0.07	0.01	0.001
%rsd	19.50	4.52	4.65	17.96
*Cerasuola*	0.192	1.519	0.108	−0.005
std. deviation	0.02	0.03	0.00	0.001
%rsd	10.89	1.65	3.59	11.95
*Nocellara*	0.209	1.536	0.111	0.003
std. deviation	0.05	0.05	0.01	0.001
%rsd	22.32	3.30	11.19	21.68

**Table 5:** Classification matrix of the olive oil based on the cultivar. The matrix contains both model and external validation samples.

Classifications: Rows (Observed) Columns (Predicted) (Analysis and Validation Samples)				
Class	Percent Correct	*Biancolilla*	*Nocellara*	*Cerasuola*
*Biancolilla*	100.00	12	0	0
*Nocellara*	100.00	0	12	0
*Cerasuola*	91.67	1	0	11
Total	97.22	13	12	11

**Table 6:** LDA tests of the significance of squared Mahalanobis distances among cultivars.

	F Tests with 5 and 24. Degrees of Freedom; Sigma-Restricted Parameterization					
Class	Biancolilla (f)	Biancolilla (p)	Nocellara (f)	Nocellara (p)	Cerasuola (f)	Cerasuola (p)
Biancolilla			156.527	0.000	89.789	0.000
Nocellara	156.527	0.000			256.103	0.000
Cerasuola	89.789	0.000	256.103	0.000		

**Table 7:** Classification matrix and test of significance of the distances of olive oils based on their harvesting period. The matrices refer to both the model and external validation samples.

(a) Classification Matrix Based on Polyphenols-Related Variables				
Class	Percent correct	harvest 1	harvest 2	
harvest 1	100	17	0	
harvest 2t	100	0	19	
Total	100	17	19	
Tests of Significance of Squared Mahalanobis Distances				
Class	harvest 1 F	harvest 1 p	harvest 2 F	harvest 2 p
harvest 1			58.612	0.000
harvest 2	58.612	0.000		
(b) Classification Matrix Based on All Continuous Variables				
Class	Percent correct	harvest 1	harvest 2	
harvest 1	100	17	0	
harvest 2	100	0	19	
Total	100	17	19	
Tests of Significance of Squared Mahalanobis Distances				
Class	harvest 1 F	harvest 1 p	harvest 2 F	harvest 2 p
harvest 1			58.612	0.000
harvest 2	58.612	0.000		

## Data Availability

The original contributions presented in the study are included in the article, further inquiries can be directed to the corresponding author.

## Abbreviations

Dehydro Oleuropein Aglycone (DHOA); European Food Safety Authority (EFSA); Extra Virgin Olive Oil (EVOO); High Resolution Mass Spectrometry (HRMS); Hydroxy-O-Decarboxymethyl Ligstroside Aglycone (HDLA); Hydroxy-O-Decarboxymethyl Oleuropein Aglycone (HDCOA); ligstroside aglycone (p-HPEA-EA); Linear Discriminant Analysis (LDA); Liquid Chromatography (LC); Methyl Oleuropein Aglycone (MOA); Oleacein (3,4-DHPEA-EDA); Oleocanthal (P-HPEA-EDA); oleuropein aglycone (3,4-DHPEA-EA);Principal Component Analysis (PCA); tyrosol (p-HPEA); hydroxytyrosol (3,4-DHPEA)
